# Stress and the HPA Axis: Role of Glucocorticoids in Alcohol Dependence

**DOI:** 10.35946/arcr.v34.4.11

**Published:** 2012

**Authors:** Mary Ann C. Stephens, Gary Wand

**Affiliations:** **Mary Ann C. Stephens, Ph.D.,***is an assistant professor in the Department of Psychiatry and Behavioral Sciences at the Johns Hopkins University School of Medicine, Baltimore, Maryland.*; **Gary Wand, M.D.,***is an Alfredo Rivière and Norma Rodriguez de Rivière Professor of Endocrinology and Metabolism and director of the Endocrine Training Program at the Johns Hopkins University School of Medicine, Baltimore, Maryland.*

**Keywords:** Alcohol dependence, problematic alcohol use, alcohol use disorders, alcohol abstinence, relapse, stress, stress response, stress hormones, hypothalamic–pituitary–adrenal axis, glucocorticoids, cortisol, brain reward pathway

## Abstract

Stress has long been suggested to be an important correlate of uncontrolled drinking and relapse. An important hormonal response system to stress—the hypothalamic–pituitary–adrenal (HPA) axis—may be involved in this process, particularly stress hormones known as glucocorticoids and primarily cortisol. The actions of this hormone system normally are tightly regulated to ensure that the body can respond quickly to stressful events and return to a normal state just as rapidly. The main determinants of HPA axis activity are genetic background, early-life environment, and current life stress. Alterations in HPA axis regulation are associated with problematic alcohol use and dependence; however, the nature of this dysregulation appears to vary with respect to stage of alcohol dependence. Much of this research has focused specifically on the role of cortisol in the risk for, development of, and relapse to chronic alcohol use. These studies found that cortisol can interact with the brain’s reward system, which may contribute to alcohol’s reinforcing effects. Cortisol also can influence a person’s cognitive processes, promoting habit-based learning, which may contribute to habit formation and risk of relapse. Finally, cortisol levels during abstinence may be useful clinical indicators of relapse vulnerability in alcohol-dependent people.

Stress, generally defined as any stimulus that disrupts the body’s internal balance (i.e., physiological homeostasis), has long been suggested to be an important correlate of uncontrolled alcohol consumption or relapse to drinking following a period of abstinence. Large epidemiological studies have reported that a variety of stressors are associated with increased alcohol consumption and binge drinking. These include hazardous and demanding work environments, legal stress, family stress (e.g., unhappy marriage and divorce), and low income (Richman et al. 1996; Rospenda et al. 2000; San Jose et al. 2000; Vasse et al. 1998). Likewise, the Health and Retirement Study found an association between stress from retirement and divorce and increased alcohol intake (Perreira and Sloan 2001). Studies also have shown that people experiencing more severe or highly threatening social stress following alcoholism treatment have higher rates of relapse compared with people not experiencing such stress (Brown et al. 1990; Noone et al. 1999). On the other hand, prospective and human laboratory studies exploring the relationship between stress, alcohol craving, and relapse have found mixed results, with more recent research suggesting that several factors moderate the effects of stress on alcohol consumption (e.g., Breese et al. 2011; Brennan et al. 1999; Fox et al. 2008; Helzer et al. 2006; Sinha 2007; Sinha and Li 2007; Thomas et al. 2011).

It remains uncertain how stress, per se, might influence vulnerability to alcohol use disorders (AUDs). However, production of the stress hormone cortisol, which is triggered by stress-induced activation of a hormonal system known as the hypothalamic–pituitary–adrenal (HPA) axis, is thought to be involved. The HPA axis is one of the main stress response pathways and has been studied extensively in relation to alcohol use (Wand 2008). Over 20 years of research has demonstrated that altered HPA axis regulation is associated with problematic alcohol use and dependence and that the nature of this dysregulation varies with respect to the stages of progression toward alcohol dependence. The finding that HPA axis dysregulation and alcohol misuse tend to co-vary has implied a “guilt-by-association” relationship—that is, that abnormal variations in stress-related cortisol production are a risk factor for developing alcoholism in the first place (Wand et al. 1993). A recent review of studies on youth and adolescents similarly suggests that HPA axis dysfunction and exposure to stress are critical components that interact to convey risk for developing AUDs (Schepis et al. 2011).

As with mood and affective disorders, many researchers consider alterations in HPA axis function crucial for understanding the underlying brain mechanisms of substance use disorders. In contrast to mood and affective disorders, however, alcohol dependence has a biphasic effect on HPA axis dynamics as a person traverses through the various phases of heavy hazardous drinking, including dependent drinking, withdrawal, abstinence, and relapse. Generally speaking, these developmental stages seem to be mirrored by a shift between hyper- and hyporesponsiveness of the HPA axis to stressful events (Rose et al. 2010). For example, hyperresponsiveness has been identified in people with a family history of alcoholism (Uhart et al. 2006; Zimmermann et al. 2004*a*,*b*), a population that is at increased risk for alcohol dependence (Windle 1997). This observation raises the question whether heightened stress responsivity is clinically meaningful to the development of alcoholism. This view is supported by studies showing that cortisol responsivity correlates with the activity of a brain system, the mesolimbic dopaminergic pathway, which is a central neural reward pathway (Oswald et al 2005; Wand et al. 2007). With transition to alcohol dependence, compensatory allostatic mechanisms result in injury to HPA axis function and elevation of stress peptide levels (e.g., corticotropin-releasing factor [CRF]) in brain regions outside the hypothalamus. The term allostasis refers to the process through which various biological processes attempt to restore homeostasis when an organism is threatened by various types of stress in the internal or external environment. Allostatic responses can involve alterations in HPA axis function, the nervous system, various signaling molecules in the body, or other systems. Allostatic alterations in HPA axis function have been posited to, among other things, injure brain reward pathways, contribute to depressed mood (i.e., dysphoria) and craving, and further contribute to the maintenance of problem drinking behavior.

This article provides an overview of the clinical evidence for HPA axis and glucocorticoid dysfunction across the developmental phases of alcoholism and explores whether this dysfunction is causally related to, or a consequence of, alcohol dependence. The article describes behavioral and physiological pathogenesis resulting from dysregulation of basal and reactive HPA axis activity. This discussion primarily focuses on human studies and studies that specifically address the glucocorticoid activation component of the stress response. The article also discusses whether these findings have potential predictive value and whether altered glucocorticoid function, regardless of etiology, may serve as a useful clinical marker for the progression of alcohol dependence and treatment prognosis. The review will not address the important role that extrahypothalamic CRF pathways play in mediating the relationship of stress and reward dysfunction (for a review of this issue, see Koob 2010).

## Physiology of the HPA Axis

**Figure 1:**
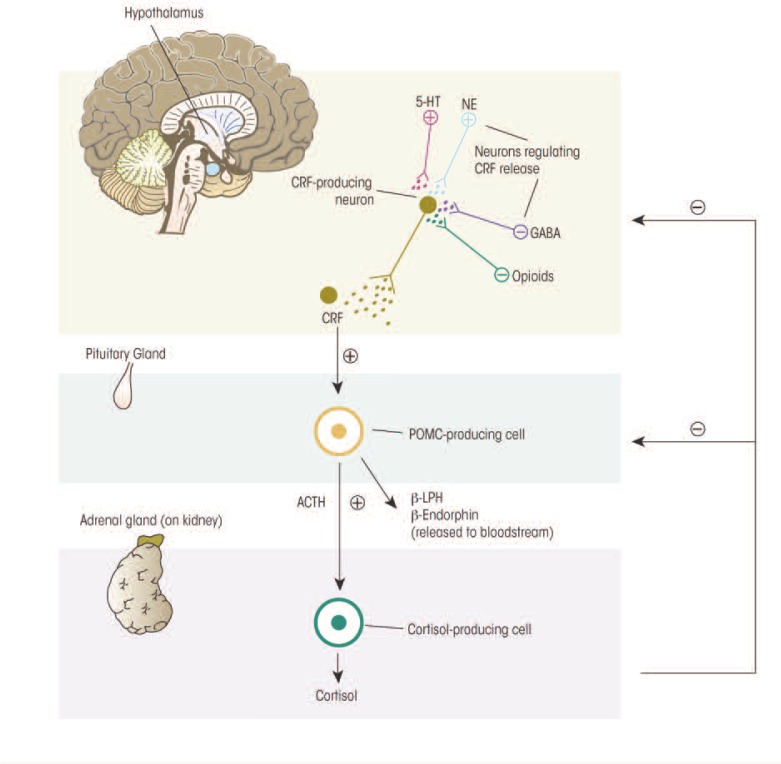
The major components of the stress response mediated by the hypothalamic–pituitary–adrenal (HPA) axis. Both alcohol and stress can induce nerve cells in one brain region (i.e., the hypothalamus) to produce and release corticotropin-releasing factor (CRF). Within the hypothalamus, CRF stimulates the release of a hormone that produces morphine-like effects (i.e., β-endorphin). CRF also is transported to a key endocrine gland, the anterior pituitary gland. There, CRF stimulates production of a protein proopiomelanocortin (POMC). POMC serves as the basis for a number of stress-related hormones, including adrenocorticotropic hormone (ACTH), β-lipotropin (β-LPH), and β-endorphin. ACTH stimulates cells of the adrenal glands to produce and release the stress hormone cortisol. When cortisol levels reach a certain level, CRF and ACTH release diminishes. Other neurons releasing serotonin (5-HT), norepin ephrine (NE), γ-aminobutyric acid (GABA), or endogenous opioids also regulate CRH release. NOTE: = ⊕ excites; ⊖ = inhibits.

The body responds to stress with self-regulating, allostatic processes aimed at returning critical systems to a set point within a narrow range of operation that ensures survival. These self-regulating processes include multiple behavioral and physiological components. Perhaps the best-studied component of the stress response in humans and mammals is activation of the HPA axis (see figure 1). Neurons in the paraventricular nucleus (PVN) of the hypothalamus release two neurohormones—CRF and arginine vasopressin (AVP)—into the blood vessels connecting the hypothalamus and the pituitary gland (i.e., hypophysial portal blood). Both hormones stimulate the anterior pituitary gland to produce and secrete adrenocorticotropic hormone (ACTH) into the general circulation. The ACTH, in turn, induces glucocorticoid synthesis and release from the adrenal glands, which are located atop the kidneys. The main glucocorticoid in humans is cortisol; the main glucocorticoid in rodents, which frequently are used as model systems to investigate the relationship between stress and alcohol use, is corticosterone. Hypothalamic activation of the HPA axis is modulated by a variety of brain signaling (i.e., neurotransmitter) systems. Some of these systems have inhibitory effects (e.g., γ-aminobutyric acid [GABA] and opioids), whereas others have excitatory effects (e.g., norepinephrine and serotonin) on the PVN. Thus, the central nervous system (CNS) and the hormone (i.e., endocrine) system are tightly interconnected to coordinate glucocorticoid activity.

To protect against prolonged activity, the HPA system is carefully modulated through negative-feedback loops designed to maintain predetermined hormone levels (i.e., set points) and homeostasis. To this end, secretion of CRF, AVP, and ACTH in part are controlled by sensitive negative feedback exerted by cortisol at the level of the anterior pituitary gland, PVN, and hippocampus. There are two types of receptors for cortisol—mineralocorticoid (type-I) and glucocorticoid (type-II) receptors—both of which participate in the negative-feedback mechanisms. Cortisol binds more strongly (i.e., has higher binding affinity) for the mineralocorticoid receptors (MRs)^1^ than the glucocorticoid receptors (GRs). Because of this difference in binding affinity, the MRs help maintain the relatively low cortisol levels circulating in the blood during the normal daily (i.e., circadian) rhythm. Only when the cortisol concentration is high (e.g., during a stressful situation) does it bind to the GRs with lower affinity; the resulting activation of the GRs terminates the stress response. This delicate negative feedback control mechanism maintains the secretion of ACTH and cortisol within a relatively narrow bandwidth. This is an extremely important homeostatic mechanism because too much or too little exposure to cortisol can have adverse consequences to health and well being.

Growing evidence suggests that a protein, FK506 binding protein 5 (FKBP5), regulates GR sensitivity. Binding of this protein to the GR reduces the receptor’s affinity for cortisol and its movement (i.e., translocation) to the nucleus. A genetic variation in FKBP5 is associated with enhanced expression of the protein following GR activation. This leads to more GR resistance, diminished negative feedback, and prolonged stress hormone activation following a stressor (Binder et al. 2004; Wochnik et al. 2005).

### Physiological Actions of Glucocorticoids

Glucocorticoids are a class of steroid hormones that are essential for the organism to survive. Cortisol, the main glucocorticoid in humans, has been placed in this class because of its effects on the metabolism of the sugar glucose, where its primary function is to increase blood glucose levels by inducing production of additional glucose molecules (i.e., gluconeogenesis). Cortisol also modifies fat and protein metabolism to support the nutrient requirements of the CNS during stress. However, cortisol also has many other wide-ranging effects when it binds to GRs. For example, it influences cardiovascular function, immunologic status (i.e., inflammatory reactions), arousal, and learning and memory; all of these systems therefore are affected when the HPA axis is activated in response to stress.^2^ Thus, cortisol helps maintain or can increase blood pressure by increasing the sensitivity of the blood vessels to signaling molecules, catecholamines. In the absence of cortisol, widening of the blood vessels (i.e., vasodilation) and hypotension occurs. The anti-inflammatory effects of cortisol are brought about by reducing proinflammatory cytokine and histamine secretion and stabilizing the membranes of cell components, lysosomes.

One of the most important actions of cortisol in the context of alcohol use and the stress response is its role in modifying learning and memory. Both stress and exposure to cortisol can transiently block memory retrieval (van Stegeren 2009), with retrieval of emotional memory more strongly affected than that of neutral memory. Of interest, both cortisol and stress also enhance memory consolidation; this process generally favors consolidation of emotionally arousing information, facilitating habit-based learning.

Consistent with the multiple-systems theory to memory organization in the mammalian CNS, studies have identified unique roles for various brain regions in learning and memory. For example, “cognitive” learning and memory is associated with activation of brain circuits in the hippocampus, whereas “habit” learning and memory is associated with activation of the dorsal striatum and the basolateral amygdala (BLA). In addition, nerve fibers projecting from the BLA modulate memory processes occurring in other brain structures. The implications of the fact that cortisol selectively affects emotionally charged memory and habit learning are discussed below.

### Determinants of HPA Axis Activity and Cortisol Exposure

Correct regulation of cortisol levels is necessary for survival, and too little or too much cortisol exposure can result in serious harm. Therefore, both basal and stress-induced cortisol levels are maintained carefully. A healthy stress response is characterized by a quick rise in cortisol levels, followed by a rapid decline with the termination of the stressful event. When the organism is burdened by cumulative stress, however, the cortisol burden increases. This results in wear and tear on the organism from excessive exposure to the catabolic properties of glucocorticoids, stress peptides, and proinflammatory cytokines. This burden taxes the organism and can influence the development of neuropsychiatric and metabolic disorders. It therefore is essential to understand the systems that regulate cortisol production.

Three main determinants of HPA axis activity control the amount of cortisol a person is exposed to during adulthood: genetic background, early-life environment, and current life stress. In addition, studies found that post-traumatic stress disorder (PTSD) can contribute to HPA axis disturbances.

#### Genetic Factors

Differences among individuals in cortisol responses to stress result from a complex interplay between genetic and environmental factors. The genetic contribution to the variability in HPA axis reactivity is believed to arise from DNA variations (i.e., polymorphisms) in the genes encoding neurotransmitters involved in HPA axis regulation. Overall, heritable influences account for approximately 62 percent of the etiological variance in basal glucocorticoid levels (Bartels et al. 2003). Recent candidate gene association studies using laboratory-based stress procedures also have implicated multiple gene variants in explaining some of the variance in cortisol responses to stress, including polymorphisms in the following genes:

- *Nr3c1*, which encodes a glucocorticoid receptor protein (Wust et al. 2004);
- *Nr3c2*, which encodes a mineralocorticoid receptor protein (DeRijk et al. 2006);
- *FKBP5* (Ising et al. 2008);
- *CRFR1*, which encodes the CRF receptor 1 protein (Clarke and Schumann 2009);
- *CRF-BP*, which encodes CRF binding protein (Wang et al. 2007);
- *GABRA6*, which encodes the GABA receptor subunit alpha-6 protein (Uhart et al. 2004);
- *OPRM1*, which encodes the mu opioid receptor protein (Chong et al. 2006); and
- *SLC6A4*, which encodes a serotonin transporter protein (Way and Taylor 2010).

It is certain that additional genes and polymorphisms will be identified in the future.

#### Early-Life Environment

Pre- and postnatal processes contribute to the lifelong responsiveness of the HPA axis to stressors. In animal models, prenatal ethanol exposure is associated with impaired HPA axis responsivity in adulthood (Hellemans et al. 2010; Weinberg et al. 2008), and emerging evidence suggests that these effects also occur in human infants and toddlers (Haley et al. 2006; Ouellet-Morin et al. 2010). Maternal stress during gestation also modifies HPA axis responsivity of infant and adult offspring (see Charil et al. 2010; Harris and Seckl 2010 for reviews). More recently, studies have focused on the consequences of early-childhood events on the stress response. Childhood trauma is a significant problem in the United States and is associated with mental and physical health problems in adulthood as well as with alterations in HPA axis function (Heim et al. 2009, 2010; Dong et al. 2004; Mangold et al. 2010). For example, it has been hypothesized that exposure to sexual and physical abuse in childhood during critical periods of brain development (i.e., during periods of neural plasticity) may permanently alter stress responsivity (Gillespie et al. 2009; Heim and Nemeroff 2001; Heim et al. 2001). Animal models that have studied this phenomenon have shown that certain forms of neonatal stress results in a modification (i.e., epigenetic methylation) of the glucocorticoid gene that has long-lasting effects on glucocorticoid responsivity (Weaver 2009). This alteration in stress responsivity may explain the observation that childhood adversity is a risk factor for the development of alcohol and other drug abuse (Epstein et al. 1998) as well as anxiety and depressive disorders in adulthood (Kessler et al. 1997; Safren et al. 2002).

Glucocorticoids also can alter the methylation patterns of other genes. For example, glucocorticoid administration to adolescent mice reduces methylation of the *FKBP5* gene in the hippocampus, hypothalamus, and blood, which is associated with enhanced expression of *FKBP5* and increased anxiety-like behavior (Lee et al. 2010). The investigators proposed that in addition to altering behaviors, methylation of the gene may be a marker of cortisol burden. Polymorphisms in *FKBP5* also have been associated with psychiatric disorders, such as depression and PTSD, that are characterized by alterations in HPA dynamics (Binder et al. 2004; Yehuda et al. 2009).

An emerging literature also addresses the role of early-childhood adversity on the development of AUDs (for a review, see Enoch 2010). For example, Schmid and colleagues (2010) found an interaction between stressful early-life events and a variant in the *CRFR1* gene that influenced age of drinking initiation and drinking progression in a population of 19-year-olds. Other studies demonstrated that certain variants of the *CRFR1* gene influenced cortisol responses to CRF and the synthetic glucocorticoid dexamethasone (Binder et al. 2010; Tyrka et al. 2009) and were associated with binge drinking in adolescents and total lifetime alcohol consumption in adults (Clarke and Schumann 2009; Hansson et al. 2006; Pastor et al. 2008; Treutlein et al. 2006). Thus, it seems that an interaction between the *CRFR1* gene and early-life events can modify HPA axis dynamics and risk for AUDs. It is certain that other stress gene variants also will be found to interact with environmental factors to increase the risk of AUDs.

#### Current Stress

Independent of prenatal and childhood stressors, periods of severe, chronic stress in adulthood, such as family- and work-related problems, combat exposure, neighborhood violence, chronic illness, or the development of neuropsychiatric disorders, alter HPA axis dynamics and increase the cortisol burden. Chronic stress triggers an allostatic shift in the normal circadian rhythm of cortisol release as well as in stress-induced cortisol levels. Thus, after chronic stress baseline cortisol levels are elevated, the body’s cortisol response to acute stress is blunted, and it takes longer for stress-induced cortisol levels to return to pre-stress levels (e.g., Juster et al. 2010; McEwen 2000; Wingenfeld et al. 2009). This allostatic injury makes the HPA axis more sensitive, resulting in higher cortisol exposure or greater cortisol burden following each stressful episode (McEwen and Gianaros 2010).

#### PTSD Symptomatology

A fourth potential determinant of HPA axis activity is the presence of PTSD symptoms. The HPA axis has been the main focus of neuroendocrine research in PTSD. In a meta-analysis of 37 studies involving people with PTSD, Meewisse and colleagues (2007) examined cortisol levels in people with PTSD and control subjects. These analyses found no differences in basal cortisol levels between the two groups; however, differences did exist under certain conditions or among certain subgroups of subjects. For example, people with PTSD had lower afternoon levels of cortisol than did control subjects, and women with PTSD had significantly lower cortisol levels than women without PTSD. The specific type of trauma experienced by a person also mattered. Thus, only people who had experienced physical or sexual abuse had significantly lower cortisol levels than control subjects. These findings highlight the complexity of the relationship between HPA axis activity and PTSD pathophysiology.

People with AUDs have a high prevalence of PTSD (Kessler et al. 1997); conversely, women with PTSD were 3.5 times more likely to develop alcoholism than women who did not report past trauma (Sartor et al. 2010). It is difficult to define whether the alterations in the HPA axis seen in people with PTSD by themselves modulate risk for alcoholism because, as discussed above, a history of childhood trauma also increases risk for developing PTSD as well as alcoholism (Binder et al. 2008; Epstein et al. 1998). Therefore, it is possible that exposure to trauma in early childhood may confer the initial insult to HPA axis regulation that later influences the interaction between PTSD and alcohol use (Yehuda et al. 2010). This view is consistent with the finding that people with a flattened cortisol response following trauma had a higher risk of developing PTSD symptoms than did those with normal cortisol levels (e.g., Aardal-Eriksson et al. 2001; Anisman et al. 2001). It remains unclear, however, whether the lower levels of circulating cortisol preceded the traumatic event (Yehuda et al. 2010).

Regardless of whether an underlying HPA axis dysregulation precedes PTSD symptomatology, evidence suggests that dysregulation occurs through increased sensitivity of the negative feedback mechanisms regulating the HPA axis, resulting in lower circulating cortisol levels. Yehuda and coworkers (2009) examined the expression of all genes active in whole-blood samples as well as cortisol levels in people with and without PSTD. This analysis identified 17 genes whose expression differed between people with and without PTSD. Several of the uniquely expressed genes are involved in HPA axis function. For example, the *FKBP5* gene, which serves as a modulator of GR sensitivity, showed reduced expression in people with PTSD, consistent with enhanced GR responsiveness. Moreover, statistical analyses found that *FKBP5* expression was predicted by cortisol levels when PTSD severity also was taken into consideration (Yehuda et al. 2009). Of interest, this profile of HPA axis dysregulation is distinct from that seen with other psychiatric disorders, such as depression (Handwerger 2009). Taken together, it seems likely that dysregulation of the HPA axis associated with PTSD interacts with epigenetic and environmental influences (Yehuda et al. 2010) and that this interaction translates into increased risk for the development of AUDs.

## The HPA Axis and Alcoholism

### HPA Axis Dynamics in People at Risk for AUDs

Altered HPA axis responsivity may be present before alcohol exerts its toxic effects on the CNS and may contribute to initial vulnerability to alcoholism. This vulnerability risk likely is a result of gene–environment interaction (Clarke et al. 2008; Schepis et al. 2011). The current state of knowledge stems from an early and large body of research suggesting that people who have alcoholic family members (i.e., who are family-history positive [FHP] for alcoholism) may be more likely to develop the disorder than those with no such family history (i.e., who are family-history negative [FHN] for alcoholism) (Windle 1997). This risk seems to be linked to abnormal HPA activity (e.g., Dai et al. 2002; King et al. 2002; Sorocco et al. 2006; Uhart et al. 2006; Wand et al. 1998, 1999*a*,*b*), although the relationships appear complex. Laboratory findings have been mixed and may depend on several factors, such as which type of stressor is used, whether basal or reactive HPA response is measured, and how cortisol is stimulated. The first studies comparing HPA axis responsivity in FHP and FHN people assessed cortisol levels in response to an agent that can block the opioid receptors (i.e., the opioid receptor antagonist, naloxone). These studies identified stronger cortisol responses to naloxone in FHP subjects than in FHN subjects (Wand et al. 1998, 1999*a*,*b*,^2001^). These findings were replicated using another opioid receptor antagonist, naltrexone (King et al. 2002). These observations are particularly interesting because they implicate the endogenous opioid system in the interaction between HPA axis activity and alcoholism risk. This signaling system not only modulates the HPA axis but also is a pharmacological target for the treatment of alcohol dependence. Other studies using a psychosocial stressor rather than a pharmacologic stimulator such as naloxone also found a stronger HPA response in FHP than in FHN subjects (Uhart et al. 2006; Zimmermann et al. 2004*a*,*b*). More recent studies among infants and toddlers with prenatal alcohol exposure who also are believed to be at increased risk for alcoholism have corroborated these latter findings in male but not female children (Haley et al. 2006; Ouellet-Morin et al. 2010). Other studies, however, found blunted HPA axis function in FHP individuals (e.g., Dai et al. 2002; Sorocco et al. 2006).

### HPA Axis Dynamics During Intoxication and Withdrawal

As with stress, acute alcohol consumption also directly and indirectly activates the HPA axis by resulting in elevated levels of glucocorticoids (Richardson et al. 2008). In fact, alcohol and other drugs of abuse have been described as a physiological stressor because they can activate the HPA axis. In social drinkers, acute doses of alcohol usually increase cortisol levels, particularly if blood alcohol levels exceed 100 mg percent (Waltman et al. 1993). At some point during the transition from social drinking to alcohol dependence and abstinence, however, the HPA axis becomes dysregulated. For example, King and colleagues (2006) found that cortisol reactivity to acute alcohol administration is attenuated in heavy, hazardous drinkers compared with light, social drinkers. This observation may be related to the general process of tolerance that emerges during heavy hazardous drinking. It is important to note that the subjects in this study were binge drinkers—which reflects a pattern of drinking frequently associated with adverse consequences—but were not alcohol dependent, suggesting that alterations in the HPA axis may begin even before dependence develops.

The onset of alcohol dependence, however, is accompanied by bouts of elevated cortisol levels in the blood (i.e., hypercortisolism) as the drinker cycles though repeated episodes of alcohol intoxication and the stress of withdrawal (Adinoff et al. 1998; Wand and Dobs 1991). This transition to alcohol dependence is accompanied by an allostatic shift in HPA axis functioning, resulting in abnormally low cortisol responsivity (Koob and Le Moal 2001). Under conditions of alcohol dependence, the allostatic load—a hypothetical measure of cumulative stress—increases and burdens the organism with excessive exposure to stress hormones and peptides as well as pro-inflammatory cytokines (McEwen 2007). Increased allostatic load has been implicated not only in AUDs and other drug use disorders but also in the development psychiatric disorders (e.g., depression), metabolic syndrome, and systemic hypertension. In the context of drug use, allostatic load not only impacts the stress response via the HPA axis but also encompasses a state of reward dysregulation. At this point, the organism constantly seeks the initial rewarding effects of the drug while tolerance to those effects develops through repeated drug self-administration. This results in a dysfunctional reward system and a maladaptive response to stress. Specifically, the allostatic alterations in cortisol responsivity may have a detrimental effect on the reward systems (Wand 2008).

### HPA Axis Dynamics During Abstinence

Wand and Dobs (1991) studied HPA axis function in alcohol-dependent subjects during the first week of abstinence following supervised alcohol withdrawal on a clinical research unit. Although the participants had modestly to highly significantly elevated cortisol levels in the urine during the withdrawal period, they also demonstrated blunted HPA axis responses to CRF, a medication that blocks cortisol production (i.e., metyrapone), and the ACTH analog cosyntropin immediately following alcohol detoxification. In fact, many of the alcohol-dependent subjects met diagnostic criteria for adrenal insufficiency. Other studies have corroborated these findings of elevated cortisol during the first week of withdrawal and also showed that cortisol levels decreased significantly over time, even plunging below the normal range (Esel et al. 2001; Keedwell et al. 2001; Majumdar et al. 1989).

Later in abstinence (i.e., at 2 to 6 weeks), alcoholics generally regain normal diurnal patterns of cortisol levels (e.g., Leggio et al. 2008). However, they may continue to exhibit a deficient cortisol response to psychosocial and pharmacological HPA axis stimulation for several months (Adinoff et al. 1998, 2005*a*,*b*; Anthenelli et al. 2001; Bernardy et al. 1996). Junghanns and colleagues (2007) compared HPA axis activity in early abstainers (i.e., mean abstinence 22 days) and long-term abstainers (i.e., mean abstinence 117 days). These investigators found that longer-abstaining people showed a stronger cortisol awakening response, another indicator of HPA axis function, implying that diurnal patterns of cortisol may begin to normalize over longer periods of abstinence. Whether regulation of the HPA axis returns completely to normal, and under what conditions, remains unknown.

Several factors may impact and moderate HPA axis recovery, including severity of withdrawal symptoms (Bernardy et al. 1996), severity and duration of dependence, comorbid childhood trauma (Schafer et al. 2010), and genetic factors underlying the individual stress response. The exact role of cortisol in HPA axis recovery is unclear. Coiro and colleagues (2007) examined the effect of exercise as a biobehavioral stressor in control subjects and alcoholics over an 8-week period. Consistent with other studies, ACTH and cortisol levels were significantly lower in alcoholics in the first month of withdrawal; by 8 weeks, however, the hormonal response had returned to normal. Interestingly, exercise itself can induce cortisol release (Beaven et al. 2010; Coiro et al. 2007; Usui et al. 2011) and has been investigated as an adjunct for smoking cessation with somewhat promising findings (Williams et al. 2010). This suggests that manipulation of cortisol levels may have therapeutic potential (see below). Indeed, determining the nature, extent, and time course of the attenuated HPA axis response during abstinence may have significant clinical relevance because low levels of basal cortisol and of the ACTH response may predict relapse to alcohol use during early abstinence (Adinoff et al. 1998; Junghanns et al. 2003, 2005; Kiefer et al. 2002).

No prospective longitudinal studies have examined HPA axis changes over longer periods of abstinence. One study of alcoholics who had been abstinent for a mean of 3.5 years found similar ACTH and cortisol responses compared with healthy controls in response to both psychological and pharmacological (i.e., opioid challenge) stressors (Munro et al. 2005). However, the study did not determine whether the alcoholics had recovered a normal level of HPA response with prolonged abstinence, whether they had had a normal response all along, or whether their lack of psychological comorbidity indicated that they were less affected by secondary characteristics related to a hyporesponsive HPA axis. Another study compared alcoholics who had relapsed with abstainers after one year and found that, contrary to findings during short-term abstinence, 1-year abstainers had significantly lower levels of cortisol (Walter et al. 2006). This suggests that the relationship between HPA axis activity and alcohol recovery is dynamic and changes as abstinence persists over time.

One major limitation of these studies is that most of the work has been conducted with male alcoholics; therefore, less is known regarding the HPA hyporesponsiveness during abstinence in females. Adinoff and colleagues (2010) focused on female alcoholics and found no differences in HPA axis activity between women who had been abstinent for 4 to 8 weeks and age-matched healthy control women. Thus, HPA axis functioning over the long term and its relationship to alcohol use and recovery remains unclear and warrants further investigation.

## Possible Roles of Cortisol in the Risk and Development of AUDs

### Cortisol’s Interaction with Dopaminergic Reward Systems

Studies in animal models have demonstrated that mesocorticolimbic dopamine pathways are involved in the brain’s reward system and that the nucleus accumbens in the ventral striatum is a critical region for mediating the rewarding effects of drugs. Virtually all drugs of abuse, including alcohol, have an impact on dopaminergic activity within this brain region (Pierce and Kumaresan 2006). Imaging studies using positron emission tomography (PET) in humans have corroborated the animal findings that drugs of abuse alter mesolimbic dopaminergic activity and have helped elucidate potential neurobiological underpinnings of drug addiction (for a review, see Martinez and Narendran 2009). These and other studies in humans have shown that mesolimbic dopamine release is correlated with the positive subjective effects of the drug (Drevets et al. 2001; Hamidovic et al. 2010; Oswald et al. 2005; Volkow et al. 2002; Wand et al. 2007). However, whereas acute alcohol administration increases synaptic dopamine activity and accumulation, chronic alcohol consumption can lead to lower-than-normal dopamine levels (i.e., a hypodopaminergic state) that may motivate the drinker to seek alcohol in order to restore the normal levels of the neurotransmitter (Volkow et al. 2007). It has been postulated that elevated levels of glucocorticoids contribute to alcohol’s reinforcing effects by enhancing modulation of the dopaminergic and subjective response to alcohol (e.g., Melis et al. 2009).

Glucocorticoids and stress interact with the dopamine reward system in ways that may increase vulnerability for developing addiction (Marinelli and Piazza 2002). For example, glucocorticoids play a critical role in the reinforcing effects of psychostimulants because surgical removal of the adrenal glands (i.e., adrenalectomy), which prevents cortisol production, decreases drug self-administration. Moreover, re-introduction of glucocorticoids at levels similar to those induced by stress reverses this effect (Deroche et al. 1997). In fact, acute stress and drugs of abuse, through different mechanisms, appear to converge upon a common pathway that modifies dopamine neuron output by enhancing long-term potentiation (LTP) of excitatory synapses (Saal et al. 2003) and long-term depression (LTD) of inhibitory synapses (Niehaus et al. 2010). However, these studies did not demonstrate that this effect directly was attributable to cortisol. Another study found that the magnitude of stress-induced cortisol release significantly correlates with mesolimbic dopamine release in the ventral striatum (Pruessner et al. 2004). Taken together, these studies suggest that cortisol may facilitate firing of dopaminergic neurons and, consequently, the reward circuitry and that this process is common with and specific to many drugs of abuse (Saal et al. 2003).

Glucocorticoids themselves also are believed to have reinforcing properties in rats as they seem to modulate self-administration of alcohol and increase brain sensitivity to other addictive drugs (e.g., stimulants and opioids) in the animals. A review by Piazza and Le Moal (1997) concluded that glucocorticoid administration at levels similar to those found in physiological stress responses had positive reinforcing effects. The investigators proposed that under natural conditions (e.g., during conflicts with other animals) the rewarding effects of the glucocorticoids might counteract the aversive effects of external aggressions, thereby allowing the animal to better cope with threatening situations. Such a mechanism may play a key role in fine-tuning an individual’s adaptation to stress and in determining reward-related behavioral pathologies. Thus, increased levels of cortisol may have reinforcing effects, acting on the brain to perpetuate behaviors (e.g., alcohol consumption) that maintain high cortisol levels.

The interactions of the stress response and the rewarding effects of drugs also have been investigated in humans. Imaging studies using PET found that higher cortisol levels in response to amphetamine administration (Oswald et al. 2005) or to a psychosocial stressor (Wand et al. 2007) were positively associated with amphetamine-induced dopamine release in the ventral striatum. Furthermore, subjects with a high cortisol response to these stimuli reported more positive subjective drug effects after amphetamine administration than did subjects with a low cortisol response (Hamidovic et al. 2010; Oswald et al. 2005; Wand et al. 2007). These studies provide evidence that cortisol may play a role in drug reinforcement through its interactions with the dopaminergic reward pathway, which may, in turn, influence vulnerability for and maintenance of alcohol and other drug use.

### Cortisol’s Effect on Cognitive Processes

LTP is a process that ultimately enhances signal transmission at the synapse. This enhanced synaptic transmission, which has been observed in a variety of neural structures, is widely considered one of the leading cellular mechanisms that underlie learning and memory (Goosens and Maren 2002). As mentioned above, LTP is enhanced by stress. Cortisol has been implicated in this phenomenon because a wide- spread system of glucocorticoid receptors is found above the hypothalamus, for example, in the limbic system, notably the hippocampus and amygdala, and in the prefrontal cortex. This section discusses the impact of glucocorticoids on some of the basic (e.g., learning, acquisition, and memory) and higher (e.g., decision-making) cognitive processes that may potentially underlie development of addictive behaviors. This discussion focuses on the regulatory actions of glucocorticoids on neural structures critically involved in cognitive processes related to alcoholism but does not cover the equally important reciprocal effects these structures have on regulating HPA axis function (e.g., Dedovic et al. 2009).

Optimal levels of cortisol are needed not only to meet the body’s physical needs but also for learning, memory, and cognitive performance. Both too little and too much cortisol may be damaging and disruptive to memory formation, whereas normal levels of glucocorticoids protect the brain against adverse events and are essential for cognitive processes. Several studies partly may explain this paradox by describing the roles of MRs and GRs in the various stages of information processing and the context in which glucocorticoid-receptor activation takes place. The effects of glucocorticoids on brain tissue as well as cognition can turn from adaptive into maladaptive when actions via both receptor types are imbalanced for a prolonged time (Joels et al. 2008; de Kloet et al. 2007).

The secretion of cortisol and norepinephrine in response to acute stress is known to affect learning and memory (Smeets et al. 2011; van Stegeren et al. 2010). The mammalian brain does not house a solitary brain region mediating the acquisition, consolidation, and retrieval of all types of learned information. Instead, memory and learning are organized in multiple brain systems. Certain brain regions (e.g., the pre-frontal cortex) govern goal-directed learning, whereas others (e.g., the dorsal striatum) are responsible for habit formation. Stress can induce a bias by promoting habit-based forms of learning and memory in lieu of goal-directed performance. Specifically, studies in rodents have determined that corticosterone and norepinephrine promote habit-based memory formation by acting on the amygdala, hippocampus, dorsal striatum, and prefrontal cortex—all of which also are involved in alcohol dependence. The relationship between cortisol and the vulnerability to alcohol dependence as well as to relapse after abstinence could involve cortisol’s effects on habit-based learning. In view of the habit-like nature of addictive behaviors, it is fascinating that recent evidence indicates a role for the habit memory system located in the dorsal striatum in the maintenance and expression of drug-seeking and drug-taking behaviors (Everitt et al. 2008). For example, anxiety-inducing (i.e., anxiogenic) drugs can promote the use of dorsal striatal-dependent habit memory in rats (Packard 2009).

Research in humans also has shown that stress is associated with decreased use of cognitive behavioral strategies, which involve the hippocampus, and increased use of stimulus-response strategies, which involve the caudate nucleus (Kim et al. 2001; Schwabe et al. 2007). It is possible that the heightened cortisol responsivity in people at increased risk for alcohol dependence may promote the transition to heavy, hazardous drinking through cortisol’s ability to promote habit-based memory formation and learning during alcohol intoxication, especially during states of heightened arousal (Smeets et al. 2009). Furthermore, the wide fluctuations in cortisol secretion observed in alcohol-dependent people could help maintain these habit-based addictive behaviors. Additionally, the hypercortisolism associated with alcohol dependence may in part promote relapse by favoring the use of habit-based memory to guide the expression of maladaptive behaviors. Finally, persistent hypercortisolism observed during repeated episodes of acute alcohol intoxication and withdrawal may be toxic to neurons in the hippocampus. Hippocampal damage, in turn, may result in alcohol-related symptoms such as personality changes, memory loss, and depression.

Chronic exposure to elevated glucocorticoid levels also can have a detrimental effect on prefrontal cortex function with concomitant neuronal degeneration (Bennett 2008). As mentioned earlier, the prefrontal cortex is involved in complex cognitive operations, including assessing likelihood of reward or punishment during critical decision-making situations as well as assessing internal and external affective cues and responding adaptively, particularly in stressful situations. Psychosocial stress can disrupt prefrontal cortex function in humans (e.g., Liston et al. 2009). However, the specific effects of glucocorticoids in this process remain to be determined (Het et al. 2005) because other physiological changes that occur as part of the overall stress response, such as increased catecholamine levels, also alter prefrontal cortex function (Qin et al. 2009). Animal studies have suggested that glucocorticoids play a role in the cognitive deficits observed after withdrawal from chronic alcohol consumption (Rose et al. 2010). In mice, the glucocorticoid receptor antagonist mifepristone reduced memory deficits during the first and second week after alcohol withdrawal, suggesting that heightened glucocorticoid levels during withdrawal directly contribute to these cognitive deficits (Jacquot et al. 2008). Studies in humans found that cognitive impairment in abstinent alcoholics was related to an attenuated cortisol response to a psychosocial stressor (Errico et al. 2002). Poorer cognitive performance also was related to more withdrawal episodes, heavier alcohol consumption, and higher cortisol levels during withdrawal (Errico et al 2002; Keedwell et al. 2001). Thus, further studies should investigate the mechanism through which altered stress regulation of the HPA axis impairs cognitive function and relates to poor prognosis in recovering alcoholics.

The amygdala is another limbic structure that is affected by cortisol in ways that might contribute to alcohol dependence. The amygdala is a major extrahypothalamic source of CRF-containing neurons that carry large numbers of CRF-1 and CRF-2 receptors; it has a primary role in the processing and memory of emotional reactions. Thus, the extended amygdala is crucial for the expression of anxiety, and the central amygdala is a major extrahypothalamic site where CRF is produced and plays a role in mediating fear and anxiety (Gray and Bingaman 1996; Heilig et al. 1994). Whereas the hypothalamic CRF system is important for modulating neuroendocrine responses to stress, the extrahypothalamic CRF system manifests the behavioral response to stress via the amygdala and other limbic regions. In rats with high alcohol preference and anxiety levels, CRF gene expression is reduced in the central nucleus of the amygdala (Hwang et al. 2004); moreover, the extracellular levels of CRF in the central amygdala are increased during acute alcohol withdrawal and during exposure to various forms of stress (Merlo-Pich et al. 1995). Chronically elevated corticosterone levels also increase CRF expression in the central amygdala (Shepard et al. 2000; Schulkin et al. 1998). This enhanced CRF production may contribute to anxiety-like behaviors. The heightened or exaggerated emotional and fearful reactivity to perceived stress, in turn, may drive alcohol consumption observed during heavy, hazardous drinking and alcohol dependence. Consistent with this theory, administration of CRF antagonists reverses anxiety-like behaviors and excessive alcohol drinking associated with alcohol withdrawal (Valdez et al. 2003). These observations suggest that heightened cortisol exposure influences alcohol consumption by inducing anxiety and dysphoria via CRF-mediated activation of the amygdala.

### Early Abstinence and Relapse

As mentioned earlier, a blunted hormonal response to stress during early abstinence is related to increased risk for relapse (Junghanns et al. 2003, 2005; Kiefer et al. 2002). The mechanism underlying this relationship is not clear. Because cortisol levels in alcohol-dependent people negatively correlate with self-reported alcohol craving (Bohn et al. 1995), it is possible that relapse to alcohol consumption during early abstinence partly is driven by alcohol’s ability to induce cortisol elevation (Junghanns et al. 2005). If this is the case, cortisol may influence the motivation to drink and relapse via a potential negative-reinforcement pathway. Several observations support this hypothesis. For example, several studies evaluating pharmacological treatments for relapse prevention during early abstinence have examined the relationships among HPA activity, craving, and alcohol intake during early abstinence, based on the hypothesis that risk for relapse may be attenuated through mechanisms that reduce craving and increase cortisol. For example, O’Malley and colleagues (2002) administered naltrexone or placebo for 6 days to alcohol-dependent, non-treatment seekers who then participated in an alcohol self-administration session. Naltrexone treatment resulted in higher cortisol levels, which were associated with lower levels of craving and less alcohol consumption. Similarly, Kiefer and colleagues (2006) studied the efficacy of naltrexone and/or an agent that can block receptors for the neurotransmitter GABA (i.e., acamprosate), both of which are used in alcoholism treatment to reduce craving. The study found that without an active treatment, both ACTH and cortisol levels decreased during early abstinence; conversely, treatment with naltrexone and acamprosate prevented these declines. Moreover, increased ACTH and cortisol during treatment was associated with reduced risk of relapse. Finally, Sinha and colleagues (2009) found that alcohol-dependent patients who had been abstinent for 28 days showed significantly elevated basal cortisol levels as well as a blunted cortisol response to a psychological stressor and to exposure to an alcohol-related cue. Further, stress and cue exposure resulted in significantly enhanced and persistent craving. Although some studies have not been able to demonstrate correlations between changes in cortisol and craving (e.g., Pratt and Davidson 2009), decreased cortisol levels in general have been accompanied by increased craving during early abstinence, which may underlie risk for relapse to alcohol use. Taken together, these studies suggest that cortisol levels and HPA axis reactivity may be useful clinical indicators in the management of relapse risk and that manipulating HPA axis regulation through either pharmacological or psychosocial intervention is a viable avenue of research for developing new alcoholism treatments.

## Summary

**Figure 2:**
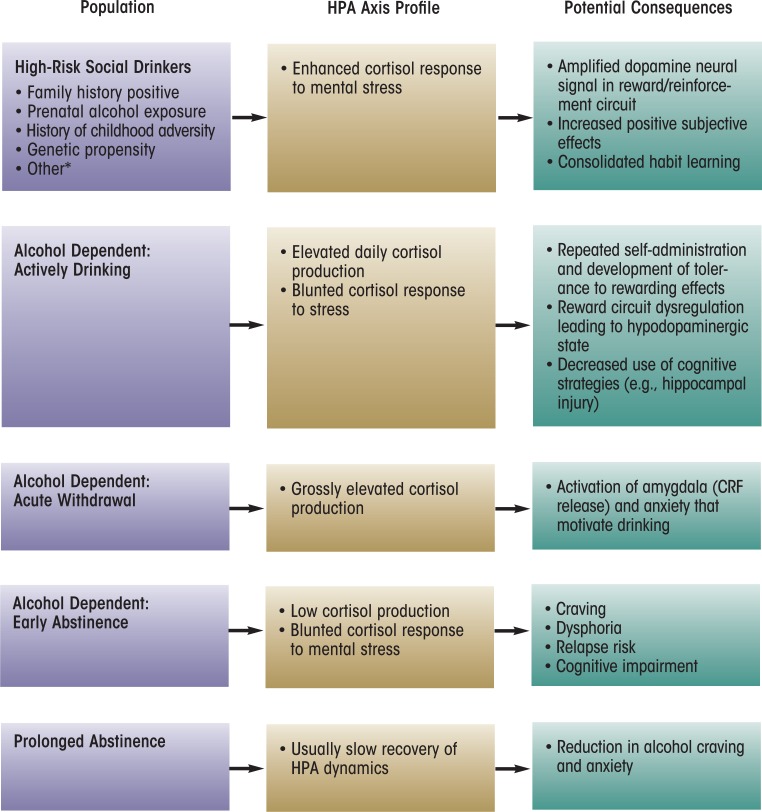
Summary of the activity of the hypothalamic–pituitary–adrenal (HPA) axis during different stages of alcoholism development and their potential consequences. NOTE: *Low level of response (LR) to alcohol is a phenotype that predicts higher risk for alcohol-related problems (Hu et al. 2005); currently, there are no data characterizing HPA axis response to mental stress in this high-risk group. Posttraumatic stress disorder (PTSD) is a complicated disorder with multiple subtypes and comorbidities; the HPA axis profile of individuals with PTSD symptomatology generally is not thought to react to mental stress with enhanced responsivity and therefore does not fit the model depicted above for other high-risk social drinkers.

The HPA axis, an important physiological stress pathway, may play a significant role in the risk and development of AUDs, and the glucocorticoid cortisol may be useful as a biomarker for HPA axis homeostatic regulation. The hormones of the HPA axis act to maintain homeostasis in the presence of stress through a variety of mechanisms. When the HPA axis becomes dysregulated, regardless of cause, deviations in cortisol reactivity result that have been associated with the progressive stages of alcoholism risk, dependence, and abstinence (see figure 2). Considerable research has been devoted to identifying potential underlying mechanisms of the HPA axis dynamics that contribute to progressive stages of alcohol dependence, and the available evidence support several of these potential mechanisms.

First, non-alcohol–dependent drinkers believed to be at risk for developing an AUD, either because of their family history or because of their hazardous drinking patterns, clearly have altered HPA axis function compared with low-risk individuals. The findings regarding the exact nature of this dysregulation (i.e., whether the HPA axis shows hyper- or hyporesponsivity) are mixed, particularly within the family-history literature. However, the equivocal results most likely are related to differences in experimental strategies used and in the levels of alcohol consumption in these drinkers (e.g., tolerance level). Nevertheless, this body of literature generally has established that cortisol responsivity serves as a risk marker for the propensity for abuse or dependence.

Second, considerable evidence supports the effect of glucocorticoids in facilitating dopamine-mediated signal transmission in the brain, which has been linked to reward pathways involved in almost all drugs of abuse. Moreover, glucocorticoids themselves have positive reinforcing properties. Conversely, reduced glucocorticoid activity seems to suppress acquisition and self-administration of drugs of abuse (Fahlke et al. 1996; Goeders and Guerin 1996). Thus, glucocorticoids appear to play a critical mediating role in the dopamine reward circuit.

Third, cortisol plays a key role in brain regions that are important for cognitive learning and memory retrieval, encoding, and consolidation. These are central processes affected by shifting hyper- and hypocortisolism throughout alcohol dependence as well as by cortisol responses to stress. It is possible that such perturbations in the HPA axis consolidate the type of habit-based learning (rather than goal-directed learning) that sustains maladaptive behaviors related to alcohol use.

Finally, deficiency in cortisol response during early abstinence is predictive of relapse to alcohol and may modulate conditions that often accompany relapse episodes, such as craving, dysphoria, and severe withdrawal symptoms. Thus, cortisol levels during abstinence may be useful clinical indicators of relapse vulnerability, and interventions that increase cortisol and decrease craving might be useful to prevent relapse.

Taken together, HPA axis function may serve as a predictor of risk for alcohol dependence in alcohol-naïve or social drinkers, facilitate initiation and maintenance of alcohol use, or serve as a predictor for risk of relapse in abstinent alcohol-dependent individuals. Using HPA axis reactivity as a predictive marker may help to identify individuals at risk for dependence or relapse prior to development of those conditions, which would allow the individuals and their treatment providers to take action and improve overall prevention and treatment efforts for AUDs.

## Footnotes

1 Cortisol has similar affinity to the MR as does the mineralocorticoid aldosterone, which helps regulate kidney function

2 Certain tissues, however, need to be protected from cortisol, such as the kidneys, colon, and placenta. In these tissues, an enzyme, 11-β hydroxysteroid dehydrogenase type II, mediates the conversion of glucocorticoids to 11-dehydro metabolites, which are inactive.

## References

[b1-arcr-34-4-468] Aardal-Eriksson E, Eriksson TE, Thorell LH (2001). Salivary cortisol, posttraumatic stress symptoms, and general health in the acute phase and during 9-month follow-up. Biological Psychiatry.

[b2-arcr-34-4-468] Adinoff B, Best SE, Ye W (2010). Adrenocortical and pituitary glucocorticoid feedback in abstinent alcohol-dependent women. Alcoholism: Clinical and Experimental Research.

[b3-arcr-34-4-468] Adinoff B, Iranmanesh A, Veldhuis J, Fisher L (1998). Disturbances of the stress response: The role of the HPA axis during alcohol withdrawal and abstinence. Alcohol Health & Research World.

[b4-arcr-34-4-468] Adinoff B, Junghanns K, Kiefer F, Krishnan-Sarin S (2005a). Suppression of the HPA axis stress-response: Implications for relapse. Alcoholism: Clinical and Experimental Research.

[b5-arcr-34-4-468] Adinoff B, Krebaum SR, Chandler PA (2005b). Dissection of hypothalamic-pituitary-adrenal axis pathology in 1-month-abstinent alcohol-dependent men, part 2: Response to ovine corticotropin-releasing factor and naloxone. Alcoholism: Clinical and Experimental Research.

[b6-arcr-34-4-468] Anisman H, Griffiths J, Matheson K (2001). Posttraumatic stress symptoms and salivary cortisol levels. American Journal of Psychiatry.

[b7-arcr-34-4-468] Anthenelli RM, Maxwell RA, Geracioti TD, Hauger R (2001). Stress hormone dysregulation at rest and after serotonergic stimulation among alcohol-dependent men with extended abstinence and controls. Alcoholism: Clinical and Experimental Research.

[b8-arcr-34-4-468] Bartels M, Van den Berg M, Sluyter F (2003). Heritability of cortisol levels: Review and simultaneous analysis of twin studies. Psychoneuroendocrinology.

[b9-arcr-34-4-468] Beaven CM, Gill ND, Ingram JR, Hopkins WG Acute salivary hormone responses to complex exercise bouts. Journal of Strength & Conditioning Research.

[b10-arcr-34-4-468] Bennett AOM (2008). Stress and anxiety in schizophrenia and depression: Glucocorticoids, corticotropin-releasing hormone and synapse regression. Australian and New Zealand Journal of Psychiatry.

[b11-arcr-34-4-468] Bernardy NC, King AC, Parsons OA, Lovallo WR (1996). Altered cortisol response in sober alcoholics: An examination of contributing factors. Alcohol.

[b12-arcr-34-4-468] Binder EB, Bradley RG, Liu W (2008). Association of FKBP5 polymorphisms and childhood abuse with risk of posttraumatic stress disorder symptoms in adults. JAMA: Journal of the American Medical Association.

[b13-arcr-34-4-468] Binder EB, Owens MJ, Liu W (2010). Association of polymorphisms in genes regulating the corticotropin-releasing factor system with antidepressant treatment response. Archives of General Psychiatry.

[b14-arcr-34-4-468] Binder EB, Salyakina D, Lichtner P (2004). Polymorphisms in FKBP5 are associated with increased recurrence of depressive episodes and rapid response to antidepressant treatment. Nature Genetics.

[b15-arcr-34-4-468] Bohn MJ, Krahn DD, Staehler BA (1995). Development and initial validation of a measure of drinking urges in abstinent alcoholics. Alcoholism: Clinical and Experimental Research.

[b16-arcr-34-4-468] Breese GR, Sinha R, Heilig M (2011). Chronic alcohol neuroadaptation and stress contribute to susceptibility for alcohol craving and relapse. Pharmacology & Therapeutics.

[b17-arcr-34-4-468] Brennan PL, Schutte KK, Moos RH (1999). Reciprocal relations between stressors and drinking behavior: A three-wave panel study of late middle-aged and older women and men. Addiction.

[b18-arcr-34-4-468] Brown SA, Vik PW, McQuaid JR (1990). Severity of psychosocial stress and outcome of alcoholism treatment. Journal of Abnormal Psychology.

[b19-arcr-34-4-468] Charil A, Laplante DP, Vaillancourt C, King S (2010). Prenatal stress and brain development. Brain Research Reviews.

[b20-arcr-34-4-468] Chong RY, Oswald L, Yang X (2006). The mu-opioid receptor polymorphism A118G predicts cortisol responses to naloxone and stress. Neuropsychopharmacology.

[b21-arcr-34-4-468] Clarke TK, Schumann G (2009). Gene-environment interactions resulting in risk alcohol drinking behaviour are mediated by CRF and CRF1. Pharmacology, Biochemistry, and Behavior.

[b22-arcr-34-4-468] Clarke TK, Treutlein J, Zimmermann US (2008). HPA-axis activity in alcoholism: Examples for a gene-environment interaction. Addiction Biology.

[b23-arcr-34-4-468] Coiro V, Casti A, Jotti GS (2007). Adrenocorticotropic hormone/cortisol response to physical exercise in abstinent alcoholic patients. Alcoholism: Clinical and Experimental Research.

[b24-arcr-34-4-468] Dai X, Thavundayil J, Gianoulakis C (2002). Response of the hypothalamic-pituitary-adrenal axis to stress in the absence and presence of ethanol in subjects at high and low risk of alcoholism. Neuropsychopharmacology.

[b25-arcr-34-4-468] de Kloet ER, Derijk RH, Meijer OC (2007). Therapy Insight: Is there an imbalanced response of mineralocorticoid and glucocorticoid receptors in depression?. Nature Clinical Practice. Endocrinology & Metabolism.

[b26-arcr-34-4-468] Dedovic K, Duchesne A, Andrews J (2009). The brain and the stress axis: The neural correlates of cortisol regulation in response to stress. NeuroImage.

[b27-arcr-34-4-468] DeRijk RH, Wust S, Meijer OC (2006). A common polymorphism in the mineralocorticoid receptor modulates stress responsiveness. Journal of Clinical Endocrinology and Metabolism.

[b28-arcr-34-4-468] Deroche V, Marinelli M, Le Moal M, Piazza PV (1997). Glucocorticoids and behavioral effects of psychostimulants. II: Cocaine intravenous self-administration and reinstatement depend on glucocorticoid levels. Journal of Pharmacology and Experimental Therapeutics.

[b29-arcr-34-4-468] Dong M, Giles WH, Felitti VJ (2004). Insights into causal pathways for ischemic heart disease: Adverse childhood experiences study. Circulation.

[b30-arcr-34-4-468] Drevets WC, Gautier C, Price JC (2001). Amphetamine-induced dopamine release in human ventral striatum correlates with euphoria. Biological Psychiatry.

[b31-arcr-34-4-468] Ehrenreich H, Schuck J, Stender N (1997). Endocrine and hemodynamic effects of stress versus systemic CRF in alcoholics during early and medium term abstinence. Alcoholism: Clinical and Experimental Research.

[b32-arcr-34-4-468] Enoch MA (2011). The role of early life stress as a predictor for alcohol and drug dependence. Psychopharmacology (Berlin).

[b33-arcr-34-4-468] Epstein JN, Saunders BE, Kilpatrick DG, Resnick HS (1998). PTSD as a mediator between childhood rape and alcohol use in adult women. Child Abuse & Neglect.

[b34-arcr-34-4-468] Errico AL, King AC, Lovallo WR, Parsons OA (2002). Cortisol dysregulation and cognitive impairment in abstinent male alcoholics. Alcoholism: Clinical and Experimental Research.

[b35-arcr-34-4-468] Errico AL, Parsons OA, King AC, Lovallo WR (1993). Attenuated cortisol response to biobehavioral stressors in sober alcoholics. Journal of Studies on Alcohol.

[b36-arcr-34-4-468] Esel E, Sofuoglu S, Aslan SS (2001). Plasma levels of beta-endorphin, adrenocorticotropic hormone and cortisol during early and late alcohol withdrawal. Alcohol and Alcoholism.

[b37-arcr-34-4-468] Everitt BJ, Belin D, Economidou D (2008). Review Neural mechanisms underlying the vulnerability to develop compulsive drug-seeking habits and addiction. Philosophical Transactions of the Royal Society of London. Series B: Biological Sciences.

[b38-arcr-34-4-468] Fahlke C, Hard E, Hansen S (1996). Facilitation of ethanol consumption by intracerebroventricular infusions of corticosterone. Psychopharmacology (Berlin).

[b39-arcr-34-4-468] Fox HC, Hong KI, Siedlarz K, Sinha R (2008). Enhanced sensitivity to stress and drug/alcohol craving in abstinent cocaine-dependent individuals compared to social drinkers. Neuropsychopharmacology.

[b40-arcr-34-4-468] Gillespie CF, Phifer J, Bradley B, Ressler KJ (2009). Risk and resilience: Genetic and environmental influences on development of the stress response. Depression and Anxiety.

[b41-arcr-34-4-468] Goeders NE, Guerin GF (1996). Effects of surgical and pharmacological adrenalectomy on the initiation and maintenance of intravenous cocaine self-administration in rats. Brain Research.

[b42-arcr-34-4-468] Gonzales RA, Job MO, Doyon WM (2004). The role of mesolimbic dopamine in the development and maintenance of ethanol reinforcement. Pharmacology & Therapeutics.

[b43-arcr-34-4-468] Goosens KA, Maren S (2002). Long-term potentiation as a substrate for memory: Evidence from studies of amygdaloid plasticity and Pavlovian fear conditioning. Hippocampus.

[b44-arcr-34-4-468] Gray TS, Bingaman EW (1996). The amygdala: Corticotropin-releasing factor, steroids, and stress. Critical Reviews in Neurobiology.

[b45-arcr-34-4-468] Haley DW, Handmaker NS, Lowe J (2006). Infant stress reactivity and prenatal alcohol exposure. Alcoholism: Clinical and Experimental Research.

[b46-arcr-34-4-468] Hamidovic A, Childs E, Conrad M (2010). Stress-induced changes in mood and cortisol release predict mood effects of amphetamine. Drug and Alcohol Dependence.

[b47-arcr-34-4-468] Handwerger K (2009). Differential patterns of HPA activity and reactivity in adult posttraumatic stress disorder and major depressive disorder. Harvard Review of Psychiatry.

[b48-arcr-34-4-468] Hansson AC, Cippitelli A, Sommer WH (2006). Variation at the rat Crhr1 locus and sensitivity to relapse into alcohol seeking induced by environmental stress. Proceedings of the National Academy of Sciences of the United States of America.

[b49-arcr-34-4-468] Harris A, Seckl J (2010). Glucocorticoids, prenatal stress and the programming of disease. Hormones and Behavior.

[b50-arcr-34-4-468] Heilig M, Koob GF, Ekman R, Britton KT (1994). Corticotropin-releasing factor and neuropeptide Y: Role in emotional integration. Trends in Neurosciences.

[b51-arcr-34-4-468] Heim C, Bradley B, Mletzko TC (2009). Effect of childhood trauma on adult depression and neuroendocrine function: Sex-specific moderation by CRH receptor 1 gene. Frontiers in Behavioral Neuroscience.

[b52-arcr-34-4-468] Heim C, Nemeroff CB (2001). The role of childhood trauma in the neurobiology of mood and anxiety disorders: Preclinical and clinical studies. Biological Psychiatry.

[b53-arcr-34-4-468] Heim C, Newport DJ, Bonsall R (2001). Altered pituitary-adrenal axis responses to provocative challenge tests in adult survivors of childhood abuse. American Journal of Psychiatry.

[b54-arcr-34-4-468] Heim C, Shugart M, Craighead WE, Nemeroff CB (2010). Neurobiological and psychiatric consequences of child abuse and neglect. Developmental Psychobiology.

[b55-arcr-34-4-468] Hellemans KG, Sliwowska JH, Verma P, Weinberg J (2010). Prenatal alcohol exposure: Fetal programming and later life vulnerability to stress, depression and anxiety disorders. Neuroscience and Biobehavioral Reviews.

[b56-arcr-34-4-468] Helzer JE, Badger GJ, Searles JS (2006). Stress and alcohol consumption in heavily drinking men: 2 years of daily data using interactive voice response. Alcoholism: Clinical and Experimental Research.

[b57-arcr-34-4-468] Het S, Ramlow G, Wolf OT (2005). A meta-analytic review of the effects of acute cortisol administration on human memory. Psychoneuroendocrinology.

[b58-arcr-34-4-468] Hu X, Oroszi G, Chun J (2005). An expanded evaluation of the relationship of four alleles to the level of response to alcohol and the alcoholism risk. Alcoholism: Clinical and Experimental Research.

[b59-arcr-34-4-468] Hwang BH, Stewart R, Zhang JK (2004). Corticotropin-releasing factor gene expression is down-regulated in the central nucleus of the amygdala of alcohol-preferring rats which exhibit high anxiety: A comparison between rat lines selectively bred for high and low alcohol preference. Brain Research.

[b60-arcr-34-4-468] Ising M, Depping AM, Siebertz A (2008). Polymorphisms in the FKBP5 gene region modulate recovery from psychosocial stress in healthy controls. European Journal of Neuroscience.

[b61-arcr-34-4-468] Jacobson SW, Bihun JT, Chiodo LM (1999). Effects of prenatal alcohol and cocaine exposure on infant cortisol levels. Development and Psychopathology.

[b62-arcr-34-4-468] Jacquot C, Croft AP, Prendergast MA (2008). Effects of the glucocorticoid antagonist, mifepristone, on the consequences of withdrawal from long term alcohol consumption. Alcoholism: Clinical and Experimental Research.

[b63-arcr-34-4-468] Joels M, Karst H, DeRijk R, de Kloet ER (2008). The coming out of the brain mineralocorticoid receptor. Trends in Neurosciences.

[b64-arcr-34-4-468] Junghanns K, Backhaus J, Tietz U (2003). Impaired serum cortisol stress response is a predictor of early relapse. Alcohol and Alcoholism.

[b65-arcr-34-4-468] Junghanns K, Horbach R, Ehrenthal D (2007). Cortisol awakening response in abstinent alcohol-dependent patients as a marker of HPA-axis dysfunction. Psychoneuroendocrinology.

[b66-arcr-34-4-468] Junghanns K, Tietz U, Dibbelt L (2005). Attenuated salivary cortisol secretion under cue exposure is associated with early relapse. Alcohol and Alcoholism.

[b67-arcr-34-4-468] Juster RP, Sindi S, Marin MF (2011). A clinical allostatic load index is associated with burnout symptoms and hypocortisolemic profiles in healthy workers. Psychoneuroendocrinology.

[b68-arcr-34-4-468] Keedwell PA, Poon L, Papadopoulos AS (2001). Salivary cortisol measurements during a medically assisted alcohol withdrawal. Addiction Biology.

[b69-arcr-34-4-468] Kessler RC, Davis CG, Kendler KS (1997). Childhood adversity and adult psychiatric disorder in the US National Comorbidity Survey. Psychological Medicine.

[b70-arcr-34-4-468] Kiefer F, Jahn H, Otte C (2006). Hypothalamic-pituitary-adrenocortical axis activity: A target of pharmacological anticraving treatment?. Biological Psychiatry.

[b71-arcr-34-4-468] Kiefer F, Jahn H, Schick M, Wiedemann K (2002). Alcohol self-administration, craving and HPA-axis activity: An intriguing relationship. Psychopharmacology (Berlin).

[b72-arcr-34-4-468] Kim JJ, Lee HJ, Han JS, Packard MG (2001). Amygdala is critical for stress-induced modulation of hippocampal long-term potentiation and learning. Journal of Neuroscience.

[b73-arcr-34-4-468] King A, Munisamy G, de Wit H, Lin S (2006). Attenuated cortisol response to alcohol in heavy social drinkers. International Journal of Psychophysiology.

[b74-arcr-34-4-468] King AC, Schluger J, Gunduz M (2002). Hypothalamic-pituitary-adrenocortical (HPA) axis response and bio-transformation of oral naltrexone: Preliminary examination of relationship to family history of alcoholism. Neuropsychopharmacology.

[b75-arcr-34-4-468] Koob GF (2010). The role of CRF and CRF-related peptides in the dark side of addiction. Brain Research.

[b76-arcr-34-4-468] Koob GF, Le Moal M (2001). Drug addiction, dysregulation of reward, and allostasis. Neuropsychopharmacology.

[b77-arcr-34-4-468] Lee HJ, Lee MS, Kang RH (2005). Influence of the serotonin transporter promoter gene polymorphism on susceptibility to posttraumatic stress disorder. Depression and Anxiety.

[b78-arcr-34-4-468] Lee RS, Tamashiro KL, Yang X (2010). Chronic corticosterone exposure increases expression and decreases deoxyribonucleic acid methylation of Fkbp5 in mice. Endocrinology.

[b79-arcr-34-4-468] Leggio L, Ferrulli A, Cardone S (2008). Relationship between the hypothalamic-pituitary-thyroid axis and alcohol craving in alcohol-dependent patients: A longitudinal study. Alcoholism: Clinical and Experimental Research.

[b80-arcr-34-4-468] Liston C, McEwen BS, Casey BJ (2009). Psychosocial stress reversibly disrupts prefrontal processing and attentional control. Proceedings of the National Academy of Sciences of the United States of America.

[b81-arcr-34-4-468] Majumdar SK, Shaw GK, Bridges PK (1989). Relationship between plasma adrenocorticotrophic hormone and cortisol concentrations in chronic alcoholic patients with depression. Drug and Alcohol Dependence.

[b82-arcr-34-4-468] Mangold D, Wand G, Javors M, Mintz J (2010). Acculturation, childhood trauma and the cortisol awakening response in Mexican-American adults. Hormones and Behavior.

[b83-arcr-34-4-468] Marinelli M, Piazza PV (2002). Interaction between glucocorticoid hormones, stress and psychostimulant drugs. European Journal of Neuroscience.

[b84-arcr-34-4-468] Martinez D, Narendran R (2010). Imaging neurotransmitter release by drugs of abuse. Current Topics in Behavioral Neurosciences.

[b85-arcr-34-4-468] McEwen BS (2000). Allostasis and allostatic load: Implications for neuropsychopharmacology. Neuropsychopharmacology.

[b86-arcr-34-4-468] McEwen BS (2007). Physiology and neurobiology of stress and adaptation: Central role of the brain. Physiological Reviews.

[b87-arcr-34-4-468] McEwen BS, Gianaros PJ (2010). Central role of the brain in stress and adaptation: Links to socioeconomic status, health, and disease. Annals of the New York Academy of Sciences.

[b88-arcr-34-4-468] Meewisse ML, Reitsma JB, de Vries GJ (2007). Cortisol and post-traumatic stress disorder in adults: Systematic review and meta-analysis. British Journal of Psychiatry.

[b89-arcr-34-4-468] Melis M, Diana M, Enrico P (2009). Ethanol and acetaldehyde action on central dopamine systems: Mechanisms, modulation, and relationship to stress. Alcohol.

[b90-arcr-34-4-468] Merlo Pich E, Lorang M, Yeganeh M (1995). Increase of extracellular corticotropin-releasing factor-like immunoreactivity levels in the amygdala of awake rats during restraint stress and ethanol withdrawal as measured by microdialysis. Journal of Neuroscience.

[b91-arcr-34-4-468] Munro CA, Oswald LM, Weerts EM (2005). Hormone responses to social stress in abstinent alcohol-dependent subjects and social drinkers with no history of alcohol dependence. Alcoholism: Clinical and Experimental Research.

[b92-arcr-34-4-468] Niehaus JL, Murali M, Kauer JA (2010). Drugs of abuse and stress impair LTP at inhibitory synapses in the ventral tegmental area. European Journal of Neuroscience.

[b93-arcr-34-4-468] Noone M, Dua J, Markham R (1999). Stress, cognitive factors, and coping resources as predictors of relapse in alcoholics. Addictive Behaviors.

[b94-arcr-34-4-468] O’Malley SS, Krishnan-Sarin S, Farren C (2002). Naltrexone decreases craving and alcohol self-administration in alcohol-dependent subjects and activates the hypothalamo-pituitary-adrenocortical axis. Psychopharmacology (Berlin).

[b95-arcr-34-4-468] Oswald LM, Wong DF, McCaul M (2005). Relationships among ventral striatal dopamine release, cortisol secretion, and subjective responses to amphetamine. Neuropsychopharmacology.

[b96-arcr-34-4-468] Ouellet-Morin I, Dionne G, Lupien SJ (2011). Prenatal alcohol exposure and cortisol activity in 19-month-old toddlers: An investigation of the moderating effects of sex and testosterone. Psychopharmacology (Berlin).

[b97-arcr-34-4-468] Packard MG (2009). Anxiety, cognition, and habit: A multiple memory systems perspective. Brain Research.

[b98-arcr-34-4-468] Pastor R, McKinnon CS, Scibelli AC (2008). Corticotropin-releasing factor-1 receptor involvement in behavioral neuroadaptation to ethanol: A urocortin1-independent mechanism. Proceedings of the National Academy of Sciences of the United States of America.

[b99-arcr-34-4-468] Perreira KM, Sloan FA (2001). Life events and alcohol consumption among mature adults: A longitudinal analysis. Journal of Studies on Alcohol.

[b100-arcr-34-4-468] Piazza PV, Le Moal M (1997). Glucocorticoids as a biological substrate of reward: Physiological and pathophysiological implications. Brain Research Brain Research Reviews.

[b101-arcr-34-4-468] Pierce RC, Kumaresan V (2006). The mesolimbic dopamine system: The final common pathway for the reinforcing effect of drugs of abuse?. Neuroscience and Biobehavioral Reviews.

[b102-arcr-34-4-468] Pratt WM, Davidson D (2009). Role of the HPA axis and the A118G polymorphism of the muopioid receptor in stress-induced drinking behavior. Alcohol and Alcoholism.

[b103-arcr-34-4-468] Pruessner JC, Champagne F, Meaney MJ, Dagher A (2004). Dopamine release in response to a psychological stress in humans and its relationship to early life maternal care: A positron emission tomography study using [11C]raclopride. Journal of Neuroscience.

[b104-arcr-34-4-468] Qin S, Hermans EJ, van Marle HJ (2009). Acute psychological stress reduces working memory-related activity in the dorsolateral prefrontal cortex. Biological Psychiatry.

[b105-arcr-34-4-468] Richardson HN, Lee SY, O’Dell LE (2008). Alcohol self-administration acutely stimulates the hypothalamic-pituitary-adrenal axis, but alcohol dependence leads to a dampened neuroendocrine state. European Journal of Neuroscience.

[b106-arcr-34-4-468] Richman JA, Flaherty JA, Rospenda KM (1996). Perceived workplace harassment experiences and problem drinking among physicians: Broadening the stress/alienation paradigm. Addiction.

[b107-arcr-34-4-468] Rose AK, Shaw SG, Prendergast MA, Little HJ (2010). The importance of glucocorticoids in alcohol dependence and neurotoxicity. Alcoholism: Clinical and Experimental Research.

[b108-arcr-34-4-468] Rospenda KM, Richman JA, Wislar JS, Flaherty JA (2000). Chronicity of sexual harassment and generalized work-place abuse: Effects on drinking outcomes. Addiction.

[b109-arcr-34-4-468] Saal D, Dong Y, Bonci A, Malenka RC (2003). Drugs of abuse and stress trigger a common synaptic adaptation in dopamine neurons. Neuron.

[b110-arcr-34-4-468] Safren SA, Gershuny BS, Marzol P (2002). History of childhood abuse in panic disorder, social phobia, and generalized anxiety disorder. Journal of Nervous and Mental Disorders.

[b111-arcr-34-4-468] San Jose B, van de Mheen H, van Oers JA (2000). Adverse working conditions and alcohol use in men and women. Alcoholism: Clinical and Experimental Research.

[b112-arcr-34-4-468] Sartor CE, McCutcheon VV, Pommer NE (2010). Posttraumatic stress disorder and alcohol dependence in young women. Journal of Studies on Alcohol and Drugs.

[b113-arcr-34-4-468] Schafer I, Teske L, Schulze-Thusing J (2010). Impact of childhood trauma on hypothalamus-pituitary-adrenal axis activity in alcohol-dependent patients. European Addiction Research.

[b114-arcr-34-4-468] Schepis TS, Rao U, Yadav H, Adinoff B (2011). The limbic-hypothalamic-pituitary-adrenal axis and the development of alcohol use disorders in youth. Alcoholism: Clinical and Experimental Research.

[b115-arcr-34-4-468] Schmid B, Blomeyer D, Treutlein J (2010). Interacting effects of CRHR1 gene and stressful life events on drinking initiation and progression among 19-year-olds. International Journal of Neuropsychopharmacology.

[b116-arcr-34-4-468] Schulkin J, Gold PW, McEwen BS (1998). Induction of corticotropin-releasing hormone gene expression by glucocorticoids: Implication for understanding the states of fear and anxiety and allostatic load. Psychoneuroendocrinology.

[b117-arcr-34-4-468] Schwabe L, Bohringer A, Wolf OT (2009). Stress disrupts context-dependent memory. Learning & Memory.

[b118-arcr-34-4-468] Schwabe L, Oitzl MS, Philippsen C (2007). Stress modulates the use of spatial versus stimulus-response learning strategies in humans. Learning & Memory.

[b119-arcr-34-4-468] Shepard JD, Barron KW, Myers DA (2000). Corticosterone delivery to the amygdala increases corticotropin-releasing factor mRNA in the central amygdaloid nucleus and anxiety-like behavior. Brain Research.

[b120-arcr-34-4-468] Sinha R (2007). The role of stress in addiction relapse. Current Psychiatry Reports.

[b121-arcr-34-4-468] Sinha R, Fox HC, Hong KA (2009). Enhanced negative emotion and alcohol craving, and altered physiological responses following stress and cue exposure in alcohol dependent individuals. Neuropsychopharmacology.

[b122-arcr-34-4-468] Sinha R, Li CS (2007). Imaging stress- and cue-induced drug and alcohol craving: Association with relapse and clinical implications. Drug and Alcohol Review.

[b123-arcr-34-4-468] Smeets T (2011). Acute stress impairs memory retrieval independent of time of day. Psychoneuroendocrinology.

[b124-arcr-34-4-468] Smeets T, Wolf OT, Giesbrecht T (2009). Stress selectively and lastingly promotes learning of context-related high arousing information. Psychoneuroendocrinology.

[b125-arcr-34-4-468] Sorocco KH, Lovallo WR, Vincent AS, Collins FL (2006). Blunted hypothalamic-pituitary-adrenocortical axis responsivity to stress in persons with a family history of alcoholism. International Journal of Psychophysiology.

[b126-arcr-34-4-468] Thomas SE, Bacon AK, Randall PK (2011). An acute psychosocial stressor increases drinking in non-treatment-seeking alcoholics. Psychopharmacology (Berlin).

[b127-arcr-34-4-468] Treutlein J, Kissling C, Frank J (2006). Genetic association of the human corticotropin releasing hormone receptor 1 (CRHR1) with binge drinking and alcohol intake patterns in two independent samples. Molecular Psychiatry.

[b128-arcr-34-4-468] Tyrka AR, Price LH, Gelernter J (2009). Interaction of childhood maltreatment with the corticotropin-releasing hormone receptor gene: Effects on hypothalamic-pituitary-adrenal axis reactivity. Biological Psychiatry.

[b129-arcr-34-4-468] Uhart M, McCaul ME, Oswald LM (2004). GABRA6 gene polymorphism and an attenuated stress response. Molecular Psychiatry.

[b130-arcr-34-4-468] Uhart M, Oswald L, McCaul ME (2006). Hormonal responses to psychological stress and family history of alcoholism. Neuropsychopharmacology.

[b131-arcr-34-4-468] Usui T, Yoshikawa T, Orita K (2011). Changes in salivary antimicrobial peptides, immunoglobulin A and cortisol after prolonged strenuous exercise. European Journal of Applied Physiology.

[b132-arcr-34-4-468] Valdez GR, Zorrilla EP, Roberts AJ, Koob GF (2003). Antagonism of corticotropin-releasing factor attenuates the enhanced responsiveness to stress observed during protracted ethanol abstinence. Alcohol.

[b133-arcr-34-4-468] van Stegeren AH (2009). Imaging stress effects on memory: A review of neuroimaging studies. Canadian Journal of Psychiatry.

[b134-arcr-34-4-468] van Stegeren AH, Roozendaal B, Kindt M (2010). Interacting noradrenergic and corticosteroid systems shift human brain activation patterns during encoding. Neurobiology of Learning and Memory.

[b135-arcr-34-4-468] Vasse RM, Nijhuis FJ, Kok G (1998). Associations between work stress, alcohol consumption and sickness absence. Addiction.

[b136-arcr-34-4-468] Volkow ND, Fowler JS, Wang GJ (2002). Role of dopamine in drug reinforcement and addiction in humans: Results from imaging studies. Behavioural Pharmacology.

[b137-arcr-34-4-468] Volkow ND, Wang GJ, Telang F (2007). Profound decreases in dopamine release in striatum in detoxified alcoholics: Possible orbitofrontal involvement. Journal of Neuroscience.

[b138-arcr-34-4-468] Walter M, Gerhard U, Gerlach M (2006). Cortisol concentrations, stress-coping styles after withdrawal and long-term abstinence in alcohol dependence. Addiction Biology.

[b139-arcr-34-4-468] Waltman C, Blevins LS, Boyd G, Wand GS (1993). The effects of mild ethanol intoxication on the hypothalamic-pituitary-adrenal axis in nonalcoholic men. Journal of Clinical Endocrinology and Metabolism.

[b140-arcr-34-4-468] Wand G (2008). The influence of stress on the transition from drug use to addiction. Alcohol Research & Health.

[b141-arcr-34-4-468] Wand G, McCaul ME, Gotjen D (2001). Confirmation that offspring from families with alcohol-dependent individuals have greater hypothalamic-pituitary-adrenal axis activation induced by naloxone compared with offspring without a family history of alcohol dependence. Alcoholism: Clinical and Experimental Research.

[b142-arcr-34-4-468] Wand GS, Zakhari S (1993). Alcohol, the hypothalamic-pituitary-adrenal axis and the hormonal tolerance. Alcohol and the Endocrine System.

[b143-arcr-34-4-468] Wand GS, Dobs AS (1991). Alterations in the hypothalamic-pituitary-adrenal axis in actively drinking alcoholics. Journal of Clinical Endocrinology and Metabolism.

[b144-arcr-34-4-468] Wand GS, Mangold D, Ali M (1999a). Adrenocorticotropin responses to naloxone in sons of alcohol-dependent men. Journal of Clinical Endocrinology and Metabolism.

[b145-arcr-34-4-468] Wand GS, Mangold D, Ali M, Giggey P (1999b). Adrenocortical responses and family history of alcoholism. Alcoholism: Clinical and Experimental Research.

[b146-arcr-34-4-468] Wand GS, Mangold D, El Deiry S (1998). Family history of alcoholism and hypothalamic optokinetic activity. Archives of General Psychiatry.

[b147-arcr-34-4-468] Wand GS, Oswald LM, McCaul ME (2007). Association of amphetamine-induced striatal dopamine release and cortisol responses to psychological stress. Neuropsychopharmacology.

[b148-arcr-34-4-468] Wang B, You ZB, Rice KC, Wise RA (2007). Stress-induced relapse to cocaine seeking: Roles for the CRF(2) receptor and CRF-binding protein in the ventral tegmental area of the rat. Psychopharmacology (Berlin).

[b149-arcr-34-4-468] Way BM, Taylor SE (2010). The serotonin transporter promoter polymorphism is associated with cortisol response to psychosocial stress. Biological Psychiatry.

[b150-arcr-34-4-468] Weaver IC (2009). Epigenetic effects of glucocorticoids. Seminars in Fetal & Neonatal Medicine.

[b151-arcr-34-4-468] Weinberg J, Sliwowska JH, Lan N, Hellemans KG (2008). Prenatal alcohol exposure: Foetal programming, the hypothalamic-pituitary-adrenal axis and sex differences in outcome. Journal of Neuroendocrinology.

[b152-arcr-34-4-468] Williams DM, Whiteley JA, Dunsiger S (2010). Moderate intensity exercise as an adjunct to standard smoking cessation treatment for women: A pilot study. Psychology of Addictive Behaviors.

[b153-arcr-34-4-468] Windle M (1997). Concepts and issues in COA research. Alcohol Health & Research World.

[b154-arcr-34-4-468] Wingenfeld K, Schulz M, Damkroeger A (2009). Elevated diurnal salivary cortisol in nurses is associated with burnout but not with vital exhaustion. Psychoneuroendocrinology.

[b155-arcr-34-4-468] Wochnik GM, Ruegg J, Abel GA (2005). FK506-binding proteins 51 and 52 differentially regulate dynein interaction and nuclear translocation of the glucocorticoid receptor in mammalian cells. Journal of Biological Chemistry.

[b156-arcr-34-4-468] Wust S, Kumsta R, Treutlein J (2009). Sex-specific association between the 5-HTT gene-linked polymorphic region and basal cortisol secretion. Psychoneuroendocrinology.

[b157-arcr-34-4-468] Wust S, Van Rossum EF, Federenko IS (2004). Common polymorphisms in the glucocorticoid receptor gene are associated with adrenocortical responses to psychosocial stress. Journal of Clinical Endocrinology and Metabolism.

[b158-arcr-34-4-468] Yehuda R, Cai G, Golier JA (2009). Gene expression patterns associated with posttraumatic stress disorder following exposure to the World Trade Center attacks. Biological Psychiatry.

[b159-arcr-34-4-468] Yehuda R, Flory JD, Pratchett LC Putative biological mechanisms for the association between early life adversity and the subsequent development of PTSD. Psychopharmacology (Berlin).

[b160-arcr-34-4-468] Zimmermann U, Spring K, Kunz-Ebrecht SR (2004a). Effect of ethanol on hypothalamic-pituitary-adrenal system response to psychosocial stress in sons of alcohol-dependent fathers. Neuropsychopharmacology.

[b161-arcr-34-4-468] Zimmermann U, Spring K, Wittchen HU (2004b). Arginine vasopressin and adrenocorticotropin secretion in response to psychosocial stress is attenuated by ethanol in sons of alcohol-dependent fathers. Journal of Psychiatric Research.

